# IL-1α Signaling Is Critical for Leukocyte Recruitment after Pulmonary *Aspergillus fumigatus* Challenge

**DOI:** 10.1371/journal.ppat.1004625

**Published:** 2015-01-28

**Authors:** Alayna K. Caffrey, Margaret M. Lehmann, Julianne M. Zickovich, Vanessa Espinosa, Kelly M. Shepardson, Christopher P. Watschke, Kimberly M. Hilmer, Arsa Thammahong, Bridget M. Barker, Amariliz Rivera, Robert A. Cramer, Joshua J. Obar

**Affiliations:** 1 Montana State University, Department of Immunology & Infectious Diseases, Bozeman, Montana, United States of America; 2 Rutgers, New Jersey Medical School, Department of Pediatrics, Center for Immunity and Inflammation, Newark, New Jersey, United States of America; 3 Geisel School of Medicine at Dartmouth, Department of Microbiology & Immunology, Hanover, New Hampshire, United States of America; 4 TGen North, Pathogen Genomics Research Division, Flagstaff, Arizona, United States of America; University of Wisconsin-Madison, UNITED STATES

## Abstract

*Aspergillus fumigatus* is a mold that causes severe pulmonary infections. Our knowledge of how *A. fumigatus* growth is controlled in the respiratory tract is developing, but still limited. Alveolar macrophages, lung resident macrophages, and airway epithelial cells constitute the first lines of defense against inhaled *A. fumigatus* conidia. Subsequently, neutrophils and inflammatory CCR2+ monocytes are recruited to the respiratory tract to prevent fungal growth. However, the mechanism of neutrophil and macrophage recruitment to the respiratory tract after *A. fumigatus* exposure remains an area of ongoing investigation. Here we show that *A. fumigatus* pulmonary challenge induces expression of the inflammasome-dependent cytokines IL-1β and IL-18 within the first 12 hours, while IL-1α expression continually increases over at least the first 48 hours. Strikingly, *Il1r1*-deficient mice are highly susceptible to pulmonary *A. fumigatus* challenge exemplified by robust fungal proliferation in the lung parenchyma. Enhanced susceptibility of *Il1r1*-deficient mice correlated with defects in leukocyte recruitment and anti-fungal activity. Importantly, IL-1α rather than IL-1β was crucial for optimal leukocyte recruitment. IL-1α signaling enhanced the production of CXCL1. Moreover, CCR2+ monocytes are required for optimal early IL-1α and CXCL1 expression in the lungs, as selective depletion of these cells resulted in their diminished expression, which in turn regulated the early accumulation of neutrophils in the lung after *A. fumigatus* challenge. Enhancement of pulmonary neutrophil recruitment and anti-fungal activity by CXCL1 treatment could limit fungal growth in the absence of IL-1α signaling. In contrast to the role of IL-1α in neutrophil recruitment, the inflammasome and IL-1β were only essential for optimal activation of anti-fungal activity of macrophages. As such, *Pycard*-deficient mice are mildly susceptible to *A. fumigatus* infection. Taken together, our data reveal central, non-redundant roles for IL-1α and IL-1β in controlling *A. fumigatus* infection in the murine lung.

## Author Summary

*Aspergillus* spp. are ubiquitous in the environment, and even though individuals are regularly exposed to fungal spores clinical invasive disease is a rare manifestation. In contrast, individuals with weakened immune systems develop severe disease, such as invasive pulmonary aspergillosis (IPA). IPA is associated with extremely poor prognoses and unacceptably high mortality rates. Knowledge gained from understanding how immunocompetent mammals control *Aspergillus* challenge will help develop new immunomodulatory strategies aimed at improving patient outcomes. It is well known that neutrophils and monocytes are crucial immune cells that act to limit fungal growth. Our work demonstrates a central role for the cytokine IL-1α in orchestrating the optimal recruitment of neutrophils and monocytes, whereas IL-1β and the inflammasome are more important in activation of anti-fungal activity of the monocytes. Moreover, our studies indicate that CCR2^+^ monocytes are required for optimal production of IL-1α in the lungs of *A. fumigatus* challenged mice. Thus, our data highlight a crucial role of the IL-1 cytokine in mediating anti-fungal immunity which might be harnessed to treat clinical cases of IPA.

## Introduction

The mold *Aspergillus fumigatus* is one of the leading causes of invasive fungal infections. It is the causative agent of severe pulmonary infections such as invasive pulmonary aspergillosis (IPA), a disease of high morbidity and mortality which affects immunocompromised individuals [1]. IPA has been a disease of growing concern over recent decades due to an increase in the immunocompromised population, specifically caused by advances in immunosuppressive drugs and organ transplantation methods as well as chemotherapy treatments in cancer patients [2]. In addition, there is increasing evidence that IPA can sporadically develop in certain immunocompetent populations [3]. Currently there are no available vaccines for *A. fumigatus* and anti-fungal drugs have a modest rate of success in limiting high mortality rates typically due to late diagnosis of IPA [1,2,4]. Moreover, the recent emergence of drug resistance has further limited treatment options in certain clinical cases and geographic areas [5,6,7].

The concentration of *Aspergillus* conidia in air samples ranges from 0.2–15 conidia/m^3^ and on a daily basis an individual can inhale hundreds of conidia [8]. In most immunocompetent individuals the conidia are typically removed from the body by physical barriers encountered within the respiratory tract. However, if the conidia escape this primary immune barrier and enter the lung, they will be removed by alveolar macrophages and other resident leukocytes, such as CCR2^+^ monocytes. Conversely, in an individual lacking a sufficient immune response, *Aspergillus* conidia are able to swell, germinate, and form hyphae, invading pulmonary tissue with the potential to disseminate systemically [9,10]. Our understanding of the inflammatory pathways necessary for an immunocompetent individual to maintain control of *A. fumigatus* while constantly being exposed to conidia is an ongoing area of investigation.

Control of *A. fumigatus* growth in the lung during invasive infection is highly dependent on rapid recruitment and activation of innate immune cells, including neutrophils [11], inflammatory monocytes [10,12], NKT cells [13], and plasmacytoid dendritic cells [14]. The importance of appropriate activation of leukocytes in the control of *A. fumigatus* is highlighted by patients and mice with chronic granulomatous disease or lacking NADPH oxidase subunits, being highly susceptible to developing IPA after *A. fumigatus* challenge [15,16,17]. Furthermore, patients who become neutropenic after chemotherapy for a bone marrow transplant are at a higher risk for developing IPA [18,19,20,21]. In the murine model of *A. fumigatus* infection, CXCR2 and its ligands are important signaling components for neutrophil recruitment [22,23,24]. In the absence of CXCR2 signaling during pulmonary *A. fumigatus* infection, there is a decrease in neutrophil recruitment along with a higher fungal burden and increased mortality rate, similar to a neutropenic model [23]. Additionally, a role for CCR2 signaling has been shown to be necessary to promote recruitment and differentiation of inflammatory monocytes from the bone marrow into CD11b^+^ dendritic cells upon *A. fumigatus* infection [10,12]. However, the exact sequence of events necessary for the expression of chemotactic molecules for optimal leukocyte recruitment has not been well elucidated.

In addition, it has been shown that polymorphisms in the Interleukin (IL)-1 gene cluster may be important in determining the susceptibility or resistance to IPA in humans [25,26]. The IL-1 gene cluster codes for two pro-inflammatory cytokines, IL-1α and IL-1β, as well as the IL-1 receptor antagonist (IL-1Ra) [27]. All three of these IL-1 family members bind to the IL-1 receptor, type I (IL-1RI). IL-1α and IL-1β enhance the immune response while IL-1Ra competitively binds to IL-1RI, thereby preventing the binding of IL-1α and IL-1β [27]. Although IL-1α and IL-1β are both pro-inflammatory cytokines within the same IL-1 cytokine family, they differ in their maturation processes. IL-1α can be released as pro-IL-1α or mature IL-1α after calpain cleavage. In either form it can actively bind to IL-1RI and mediate downstream signaling [27,28]. Conversely, IL-1β is first produced as inactive pro-IL-1β which must be cleaved by a caspase-1 containing inflammasome to yield the mature biologically active cytokine [29]. After fungal exposure, IL-1β production has been linked to activation of the NLRP3 inflammasome [30,31,32,33,34,35,36,37]. Mice lacking the NLRP3 inflammasome are highly susceptible to disseminated candidiasis [30,36,37]. However, the role of inflammasome activation by *A. fumigatus in vivo* is unknown.

The role of IL-1α in regulating the pulmonary inflammatory response after infectious challenge is much less understood and is an active area of research. Importantly, several studies have shown that IL-1α and IL-1β can have non-redundant roles in infection and inflammation. Specifically, it has been demonstrated that an increase of IL-1α correlated with early neutrophil recruitment, while IL-1β correlated with macrophage recruitment during later time points in a model of sterile inflammation [38,39]. During pulmonary *Legionella pneumophila* infection IL-1α is essential for early neutrophil responses [40]. In a systemic candidiasis model, IL-1α and IL-1β played non-redundant roles in anti-fungal immunity by enhancing anti-fungal activity of leukocytes and recruitment of neutrophils, respectively [41]. However, the role(s) of IL-1 cytokines after challenge with the mold *A. fumigatus* remains to be fully defined.

Here, we delineate the differential roles of IL-1α and IL-1β after *in vivo* challenge with *A. fumigatus* and further define the sequence of events required for leukocyte recruitment after *A. fumigatus* challenge. Specifically, we observed, unlike the diseases caused by the yeast *Candida albicans* [30,36,37], that the inflammasome is not essential for preventing severe invasive pulmonary aspergillosis, but does participate in initiating the full anti-fungal activity of leukocytes. In stark contrast, IL-1α signaling through IL-1RI is crucial for the control of pulmonary *A. fumigatus* infection through optimal leukocyte recruitment, which correlated with CXCL1 expression. CCR2^+^ monocytes regulated the early expression of IL-1α and CXCL1, and promoted early neutrophil accumulation in the airways. Treatment of *Il1r1*-deficient mice with a chemokine known to enhance neutrophil recruitment enhanced immunity against pulmonary *A. fumigatus* infection. Thus, our studies define the specific sequence of events regulated by both IL-1α and IL-1β necessary for control of *A. fumigatus* growth and lung damage within the respiratory tract.

## Results

### Differential temporal expression kinetics of IL-1 cytokine family members after *Aspergillus fumigatus* challenge

**Figure 1:**
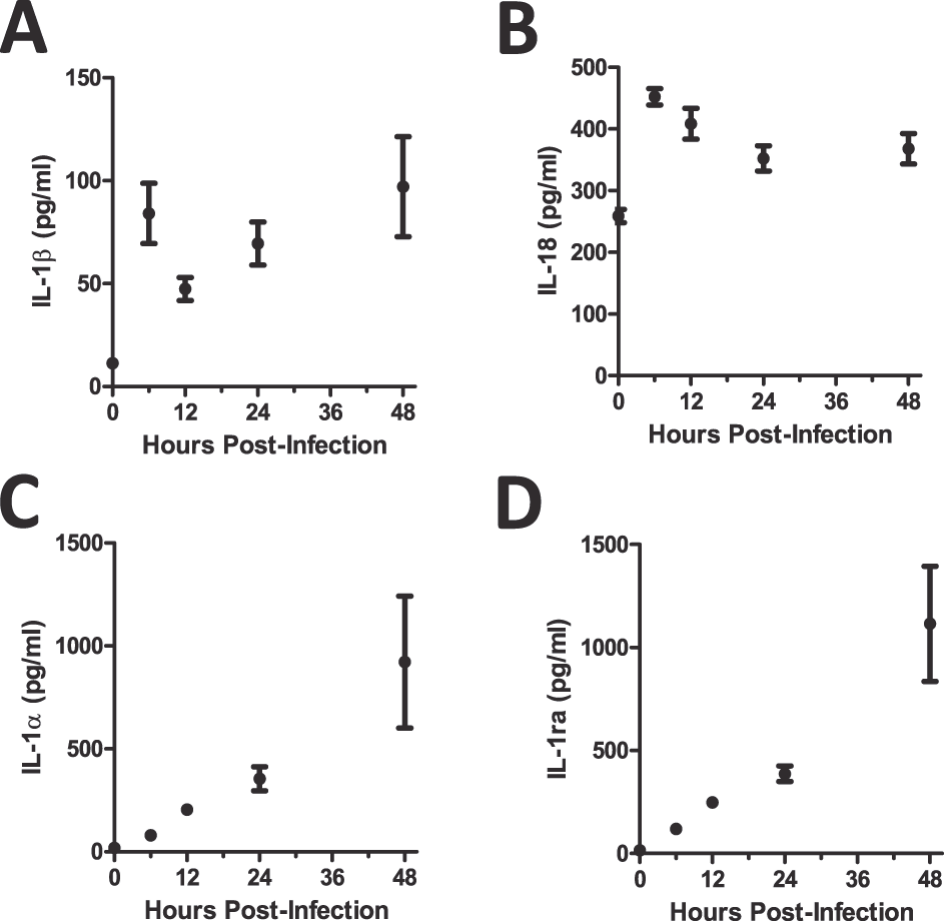
C57BL/6 mice show differential expression of IL-1α and IL-1ß after *A. fumigatus* infection. Mice were infected i.t. with 5×10^7^ CEA10 conidia and at indicated time-points, mice were euthanized, bronchoalveolar lavage fluid (BALF) collected, and lung tissue homogenized. IL-1β **(A)**, IL-18 **(B)**, IL-1α **(C)**, and IL-1Ra **(D)** levels in lung homogenate (IL-1α) and BALF (IL-1β, IL-18, and IL-1Ra) were measured using ProcartaPlex Mouse Cytokine & Chemokine 36-plex Immunoassay or ELISA. Data are representative of four mice per time point and two independent experiments. Each dot represents the mean ± one SEM.

To examine the early pulmonary inflammatory milieu induced after *A. fumigatus* challenge, bronchoalveolar lavage fluid (BALF) was collected 6, 12, 24, and 48 h after intratracheal (i.t.) instillation of ∼5×10^7^ conidia of the CEA10 strain of *A. fumigatus*. Of note, both the inflammasome-dependent cytokines IL-1β (Fig. 1A) and IL-18 (Fig. 1B) were expressed within approximately 6 h after *A. fumigatus* challenge. In contrast, IL-1α (Fig. 1C) and IL-1Ra (Fig. 1D) were expressed in a linearly increasing manner during the first 48 h. Thus, *A. fumigatus* challenge results in temporally distinct expression of IL-1 cytokine family members.

### Il1r1-deficient mice are highly susceptible to pulmonary *Aspergillus fumigatus* infection

**Figure 2:**
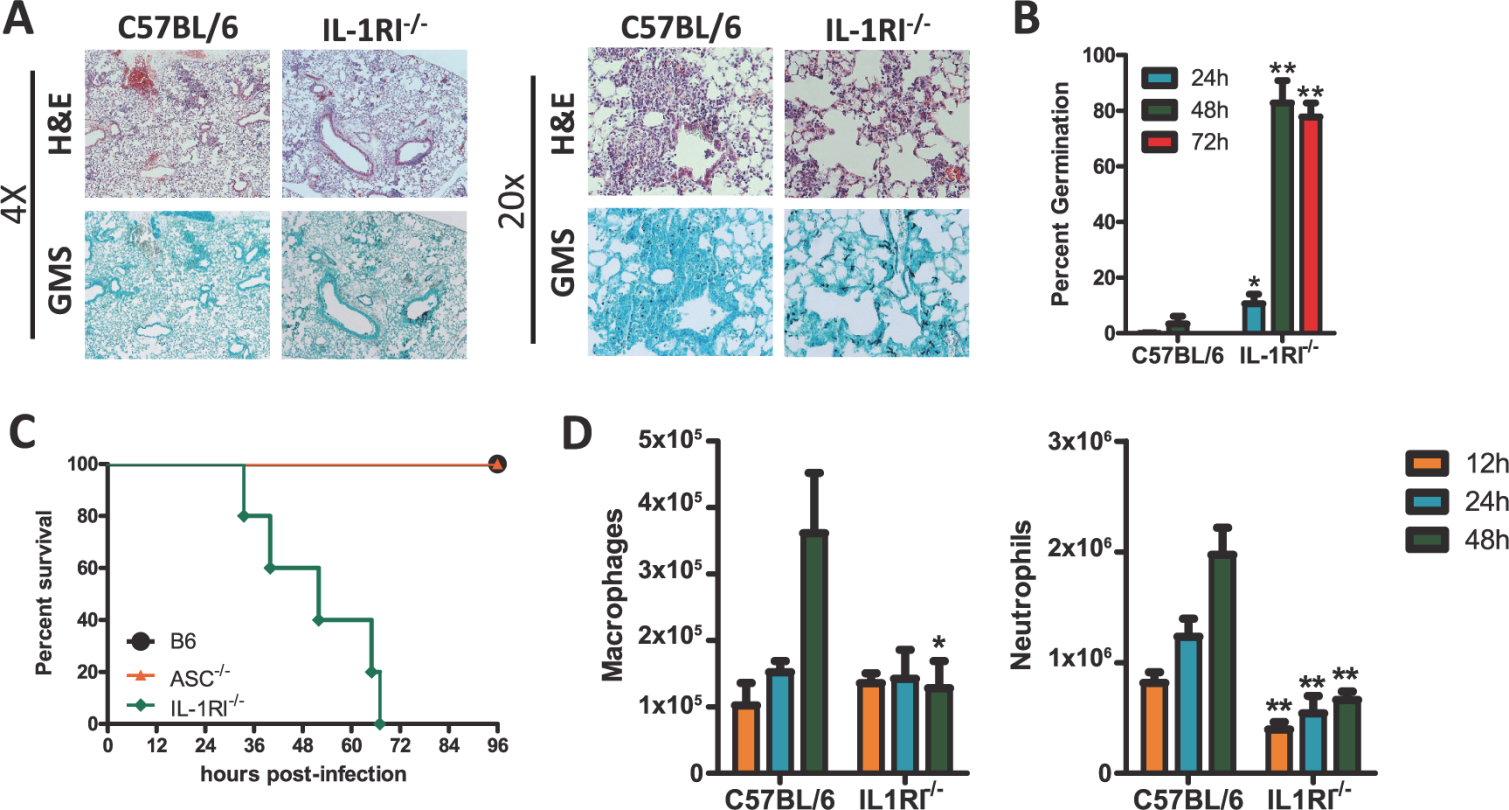
*Il1r1*-deficient mice are highly susceptible to *Aspergillus fumigatus* infection. Age-matched C57BL/6 or *Il1r1*-deficient mice were infected i.t. with 5×10^7^ CEA10 conidia and at indicated time-points, mice were euthanized, BALF collected, and lungs saved for histological analysis. **(A)** Formalin-fixed lungs were paraffin embedded, sectioned, and stained with H&E (top) or GMS (bottom) for analysis by microscopy. Representative lung sections from C57BL/6 and *Il1r1*-deficient mice infected with CEA10 for 48 h are shown using either the 4× (left) or 20× (right) objectives. **(B)** *A. fumigatus* germination rates were assessed over the first 72 h of infection by microscopically counting both the number of conidia and number of germlings in GMS-stained section. **(C)** Survival of C57BL/6, *Pycard* ^−/−^, and *Il1r1^−/−^* mice challenged i.t. with 1.5×10^7^ *A. fumigatus* (CEA10) conidia was then monitored for survival over the first 96 h (Mantel-Cox log-rank test, p = 0.0002). Data are representative of 2 independent experiments at each time point consisting of at least 5 mice per group. **(D)** Total macrophage (left panel) and neutrophil (right panel) recruitment in the BALF was measured at 12, 24, and 48 h post-infection. Data are representative of at least 2 independent experiments at each time point consisting of 3–5 mice per group. Bar graphs show the group mean ± one SEM. Statistically significant differences were determined using Student’s t-test (*p < 0.05; **p < 0.01).

In one cohort of human patients it has been shown that a complex polymorphism in the *Il1a, Il1b*, and *Il1rn* genes, which was associated with decreased IL-1 dependent inflammatory events, resulted in increased risk for the development of IPA [25]. Because of this prior clinical observation plus our finding that both IL-1α and IL-1β are produced in the lungs after *A. fumigatus* challenge (Fig. 1), we questioned whether IL-1RI signaling was critical in the clearance of *A. fumigatus* from the lung. To globally test the role of IL-1 signaling in limiting *A. fumigatus* growth in the respiratory tract of mice, we challenged C57BL/6 and *Il1r1*-deficient mice with ∼5×10^7^ conidia of *A. fumigatus* CEA10 delivered via the i.t. route. Subsequently, control of *A. fumigatus* in the respiratory tract was assessed by histological analysis at 24, 48, and 72 h after instillation. Strikingly, Grocott-Gomori methenamine silver (GMS) staining of lung tissue from *Il1r1*-deficient mice revealed the presence of a significant fraction of germinating *A. fumigatus* conidia at 48 h that was not observed in C57BL/6 mice (Fig. 2A). When the presence of germinating *A. fumigatus* conidia was quantified over the first 72 h, C57BL/6 mice displayed minimal germination that was ∼4% at 48 h before resolving (Fig. 2B); in contrast, *Il1r1*-deficient mice displayed a significant impairment in controlling *A. fumigatus* germination within 24 h (Fig. 2B). By 48 h, the majority of fungal conidia in *Il1r1*-deficient mice were germinated (Fig. 2B). High levels of germination were observed in the majority of *Il1r1*-deficient mice and this was associated with significant mortality in those mice (Fig. 2C). To strengthen our conclusion that IL-1RI signaling was crucial for controlling *A. fumigatus* germination in the lungs rather than a development issue in the *Il1r1*-deficient mice, we treated C57BL/6 mice intraperitoneally (i.p.) with 200 μg of hIL1ra, which antagonizes IL-1α and IL-1β, or placebo every 24 h starting one day prior to challenging mice with ∼5×10^7^ conidia of *A. fumigatus*. Lung tissue from hIL1ra-treated C57BL/6 mice revealed the presence of a significant fraction of germinating *A. fumigatus* conidia at 48 h, which was not observed in placebo treated C57BL/6 mice (S1 Fig.). Taken together, these results strongly support the conclusion that IL-1RI signaling is critical for prevention of *A. fumigatus* strain CEA10 pulmonary proliferation and host damage.

Neutrophils and macrophages are widely acknowledged to be critical effector cells for clearing *A. fumigatus* from the lungs [42]. Assessing cellular recruitment via differential microscopic counting of cytospins stained with Diff-Quik from the bronchoalveolar lavage fluid at 12, 24, and 48 h post-challenge demonstrated a significant impairment in neutrophil recruitment at each time point analyzed, while macrophage recruitment was similar between C57BL/6 and *Il1r1*-deficient mice at early time points after *A. fumigatus* challenge, but were decreased by 48 h (Fig. 2D). When inflammatory infiltrates within the BALF and lung parenchyma were assessed at 12, 24, and 36 h by flow cytometry a similar decrease in neutrophils in both compartments was observed in the *Il1r1*-deficient mice (S2A-S2B Fig.), while CD11b^+^ macrophages (S2C Fig.), CD11c^+^ alveolar macrophages (S2D Fig.), and CD103^+^ dendritic cells (S2E Fig.) were found at similar levels as observed in C57BL/6 mice. We next questioned whether leukocyte recruitment was diminished in *Myd88*-deficient mice because it is the key signaling adapter for IL-1RI, as well as TLRs [27,43], and *Myd88*-deficient mice have an impaired ability to control pulmonary *A. fumigatus* growth [44]. Indeed, *Myd88*-deficient mice exhibited defective neutrophil recruitment 12 and 24 h after *A. fumigatus* instillation, but normal macrophage recruitment at these early time points (S3 Fig.). Thus, mice deficient in *Il1r1* and *Myd88* are highly impaired in their ability to clear *A. fumigatus* from the lungs, which correlates with defects in early neutrophil recruitment to the lungs.

### Pycard-deficient mice are only mildly susceptible to pulmonary *Aspergillus fumigatus* exposure

**Figure 3:**
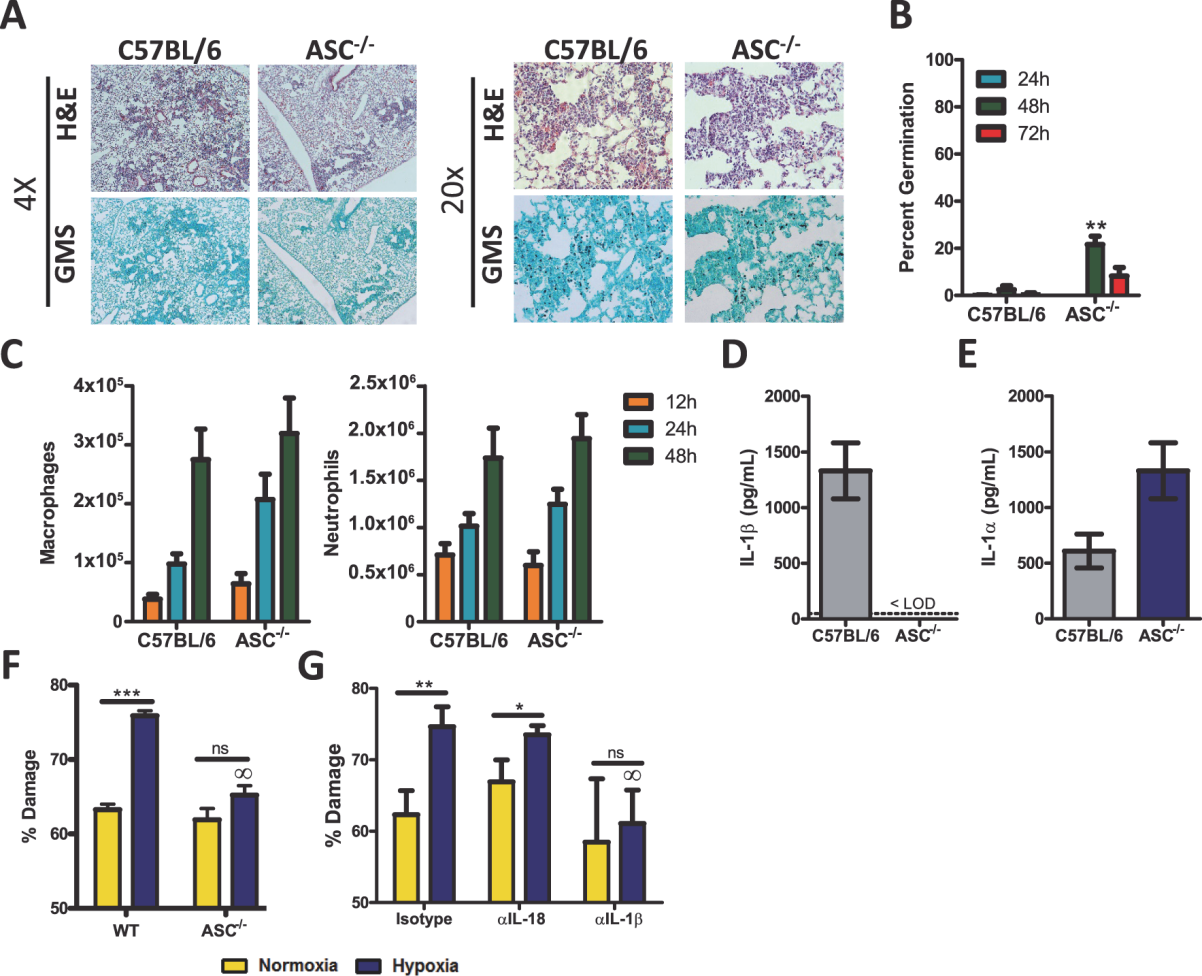
*Pycard*-deficient mice are mildly susceptible to *A. fumigatus* infection. Age-matched C57BL/6 or *Pycard*-deficient mice were infected i.t. with 5×10^7^ CEA10 conidia and at indicated time-points mice were euthanized, BALF collected, and lungs saved for histological analysis. **(A)** Formalin-fixed lungs were paraffin embedded, sectioned, and stained with H&E (top) or GMS (bottom) for analysis by microscopy. Representative lung sections from C57BL/6 and *Pycard*-deficient mice infected with CEA10 for 48 h are shown using either the 4× (left) or 20× (right) objectives. **(B)** *A. fumigatus* germination rates were assessed over the first 72 h of infection by microscopically counting both the number of conidia and number of germlings in GMS-stained section. **(C)** Total macrophage (left panel) and neutrophil (right panel) recruitment in the BALF was measured at 12, 24, and 48 h post-infection. **(D)** IL-1β levels in the bronchoalveolar lavage fluid (BALF) and **(E)** IL-1α levels in the lung parenchyma were assessed from C57BL/6 and *Pycard*-deficient mice infected i.t. 12 h prior with 5×10^7^ CEA10 conidia. IL-1α and IL-1β levels in BALF were measured by ELISA. **(B-E)** All data are representative of at least 2 independent experiments at each time point consisting of 3–5 mice per group. Bar graphs show the group mean ± one SEM. Statistically significant differences were determined using Student’s t-test (*p < 0.05; **p < 0.01). LOD = limit of detection. **(F-G)** The anti-fungal activity of bone marrow-derived macrophages (BMDM) were assessed *in vitro* using the previously described XTT assay [46]. **(E)** BMDMs were obtained from C57BL/6 (WT) or *Pycard*-deficient (ASC) mice. An XTT assay was performed using BMDM from each mouse strain in both normoxic and hypoxic conditions. **(B)** BMDMs from C57BL/6 mice were obtained and incubated with isotype control antibody, IL-18 neutralizing antibody or IL-1ß neutralizing antibody. These BMDMs were then used in an XTT assay in both normoxic and hypoxic conditions. **(E-F)** Data are representative of four biological replicates in each group. Bar graphs show the group mean ± one SEM. Statistically significant differences were determined using an one-way ANOVA with Bonferroni’s post-test (∞p < 0.05 compared to C57BL/6 cells under the same conditions).

It is well documented that IL-1β secretion requires the function of the inflammasome [29] and that both the inflammasome and IL-1β are important in limiting systemic fungal infections [30,33,35,36,37,41,45]. Recent *in vitro* studies have shown that the NLRP3-ASC-Capase1 inflammasome can be triggered by *A. fumigatus* [31], but the *in vivo* relevance of this triggering during *A. fumigatus* infection remains unknown. Multiple inflammasome complexes exist, but ASC (*Pycard*) is a central adapter protein needed for maturation of IL-1β and IL-18 [29]. Thus, to determine the role of the inflammasome after *A. fumigatus* challenge, we challenged C57BL/6 and *Pycard*-deficient mice with ∼5×10^7^ conidia of *A. fumigatus* CEA10 i.t.; subsequently, control of *A. fumigatus* in the respiratory tract was assessed by histological analysis at 24, 48, and 72 h after instillation. GMS staining of lung tissue from *Pycard*-deficient mice revealed the presence of germinating *A. fumigatus* conidia at elevated frequencies compared to C57BL/6 mice at 48 h (Fig. 3A-B), but this phenotype was less severe than what was observed in *Il1r1*-deficient mice and did not result in murine mortality (Fig. 2). When the presence of germinating *A. fumigatus* conidia was quantified over the first 72 h, C57BL/6 mice display minimal germination that was ∼3% at 48 h (Fig. 3B). *Pycard*-deficient mice displayed normal control of *A. fumigatus* germination at 24 h. However, by 48 h impaired control of *A. fumigatus* germination (∼22%) was observed, but these mice were ultimately able to resolve the *A. fumigatus* challenge (Fig. 3B). Because, neutrophils and macrophages are widely acknowledged to be critical effector cells for clearing *A. fumigatus* from the lungs [42] and were diminished in the absence of IL-1RI signaling (Fig. 2C), we next assessed inflammatory cell recruitment in BALF via differential microscopic counting of cytospins stained with Diff-Quik from the bronchoalveolar lavage fluid at 12, 24, and 48 h after instillation. Interestingly, C57BL/6 and *Pycard*-deficient mice demonstrated equivalent neutrophil and macrophage recruitment at each time point analyzed (Fig. 3C). Moreover, when the inflammatory infiltrates within the BALF and lung parenchyma were assessed at 12, 24, and 36 h by flow cytometry the number of neutrophils in the BALF and lung parenchyma, CD11b^+^ macrophages, CD11c^+^ alveolar macrophages, and CD103^+^ dendritic cells in the lung parenchyma were found at similar levels in C57BL/6 and *Pycard*-deficient mice (S2 Fig.). When we examined the expression of IL-1β in the BALF of *Pycard*-deficient mice, no expression of IL-1β at 12 h was observed while significant levels were detected in C57BL/6 mice (Fig. 3D); however, when IL-1α was examined in the lung parenchyma we observed equivalent levels of cytokine expression (Fig. 3E), suggesting that IL-1α signaling could still be activated in the *Pycard*-deficient mice.

Since *Pycard*-deficient mice did not demonstrate impaired leukocyte recruitment after *A. fumigatus* challenge, we next sought to quantitate the anti-fungal activity of macrophages from C57BL/6 and *Pycard*-deficient mice. Hyphal damage induced by macrophages was assessed using the XTT hyphal damage assay, which measures fungal cell metabolic activity as an indirect measure of fungal viability [46]. C57BL/6 and *Pycard*-deficient bone marrow-derived macrophages induced similar hyphal damage when co-cultured with *A. fumigatus* under normoxic conditions (Fig. 3F, yellow bars). Interestingly, a previous report demonstrated enhanced anti-fungal activity of leukocytes against fungal hyphae under hypoxic conditions [46], which occurs within the lungs after *A. fumigatus* challenge and at sites of microbial infection [47,48]. Intriguingly, time-points when hypoxia is observed also coincides with the recruitment of inflammatory monocytes to the site of infection [12]. Thus, we sought to test the contribution of the inflammasome to the anti-fungal response of macrophages under hypoxic conditions. Similar to the previous findings [46], C57BL/6 bone marrow-derived macrophages displayed significantly enhanced anti-fungal activity when cultured in hypoxia (Fig. 3F). In contrast, *Pycard*-deficient macrophages induced less hyphal damage when co-cultured with *A. fumigatus* under hypoxic conditions (Fig. 3F, blue bars). Since activation of the inflammasome triggers the release of both IL-1β and IL-18 we next sought to assess which inflammasome-dependent cytokine was responsible for increasing the anti-fungal activity of macrophages in hypoxia. C57BL/6 macrophages were treated with an isotype control antibody, anti-IL1β antibody, or anti-IL18 antibody during the co-culture with *A. fumigatus* germlings. Subsequently, fungal damage was again assessed by an XTT assay. Macrophages treated with an isotype control antibody display increased anti-fungal activity in hypoxia. This increased anti-fungal activity was lost in the presence of a blocking anti-IL1β antibody, but not a blocking anti-IL18 antibody (Fig. 3G). Collectively, these data demonstrate that mice deficient in *Pycard* are mildly impaired in their ability to clear *A. fumigatus* from the lungs, which correlated with *in vitro* defects in the anti-hyphal activity induced by IL-1β in hypoxia, rather than inflammatory cell recruitment to the lungs.

### Anti-IL-1α treatment impairs pulmonary recruitment of leukocytes, enhancing the susceptibility of mice to pulmonary *Aspergillus fumigatus* infection

**Figure 4:**
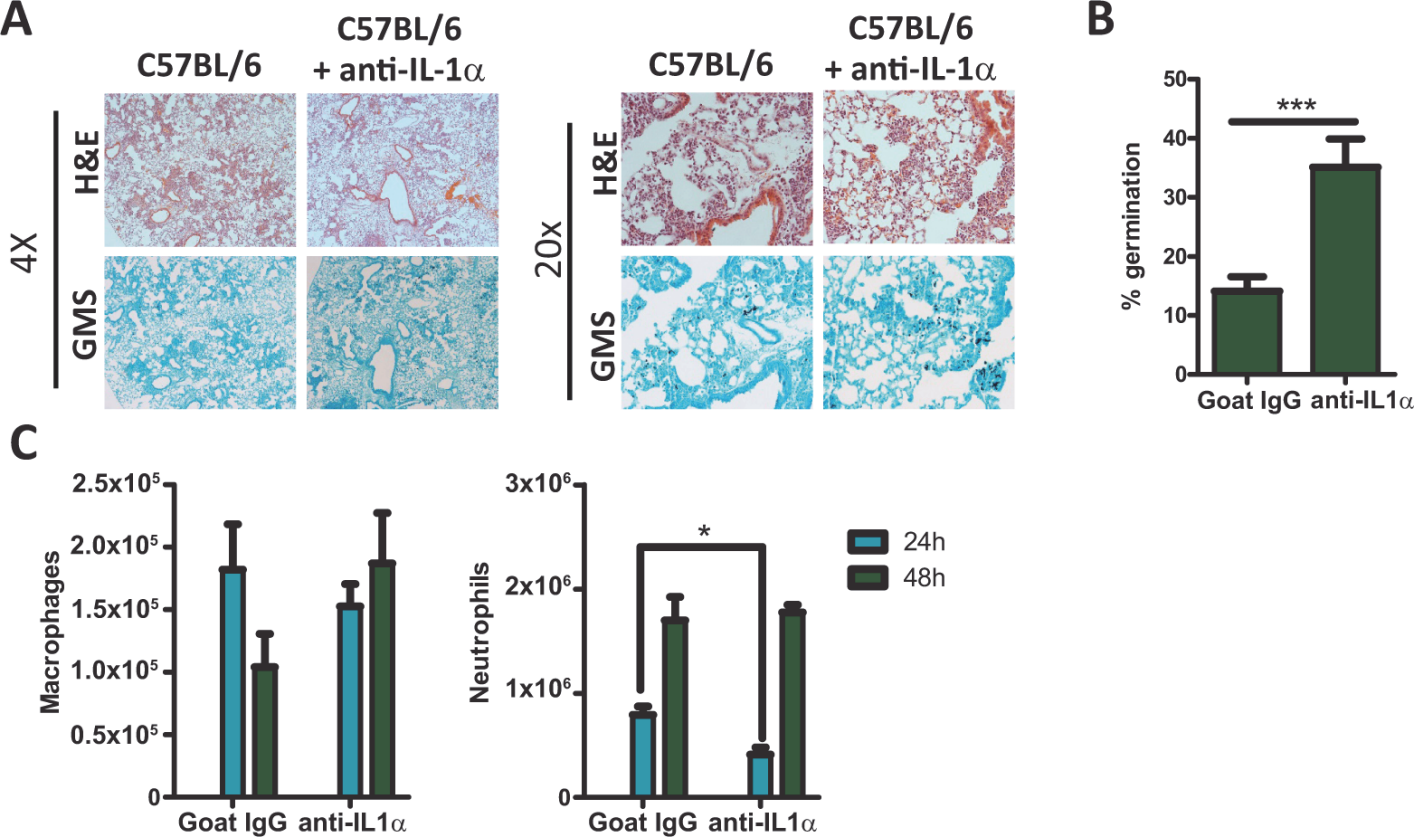
C57BL/6 mice treated with IL-1α neutralizing antibody were more susceptible to *Aspergillus fumigatus* infection. C57BL/6 mice treated with isotype control antibody or IL-1α neutralizing antibody were infected i.t. with 5×10^7^ CEA10 conidia. At the indicated time points mice were euthanized, BALF collected and lungs saved for histological analysis. **(A)** Formalin-fixed lungs were paraffin embedded, sectioned and stained with H&E (top) or GMS (bottom) for analysis by microscopy. Representative lung sections from C57BL/6 mice treated with isotype control antibody (left) or with anti-IL-1α antibody (right) and infected with CEA10 for 48 h are shown using either the 4× (left) or 20× (right) objectives. **(B)** *A. fumigatus* germination rates at 48 h after challenge was determined by microscopically counting both the number of conidia and number of germlings in GMS-stained section. **(C)** Total macrophage (left panel) and neutrophil (right panel) recruitment in the BALF was measured at 24 and 48 h post-infection via cytospins. Data are representative of two independent experiments consisting of 4–5 mice per group. Bar graphs show the group mean ± one SEM. Statistically significant differences were determined using a Student’s t-test (*p < 0.05, ***p < 0.001).

As *Il1r1*-deficient mice were much less able to control *A. fumigatus* germination than *Pycard*-deficient mice (Fig. 2 & 3) and *Pycard*-deficient mice still produced IL-1α in the lungs (Fig. 3D), we next sought to understand the role IL-1α played in the clearance of *A. fumigatus* from the lung. To determine the role of IL-1α after *A. fumigatus* challenge, we treated C57BL/6 mice i.p. with 40 μg of goat IgG or anti-IL1α 24 h prior to and 24 h after challenging mice with ∼5×10^7^ conidia of *A. fumigatus*. Control of *A. fumigatus* in the respiratory tract was assessed by histological analysis at 48 h after instillation. GMS staining of lung tissue from anti-IL1α treated C57BL/6 mice revealed the presence of germinating *A. fumigatus* conidia at significantly higher frequencies than seen in goat IgG treated C57BL/6 mice (Fig. 4A & B). As leukocyte recruitment to the lungs was significantly impaired in *Il1r1*-deficient, but not *Pycard*-deficient, mice following *A. fumigatus* challenge, we next assessed inflammatory cell recruitment to the BALF via differential microscopic counting of cytospins stained with Diff-Quik from the bronchoalveolar lavage fluid at 24 and 48 h post-challenge. Interestingly, anti-IL1α treated C57BL/6 mice demonstrated reduced neutrophil recruitment at 24 h post-*A. fumigatus* challenge (Fig. 4C). Additionally, treatment of *Pycard*-deficient mice with anti-IL1α significantly enhanced the susceptibility of those mice to *A. fumigatus* challenge, mirroring what was found in *Il1r1*-deficient mice (S4 Fig.). Thus, blocking IL-1α in mice significantly impairs early neutrophil recruitment to the lungs early after *A. fumigatus* challenge resulting in impaired control of *A. fumigatus* germination in the lungs.

### IL-1α signaling enhances the expression of CXCL1

**Figure 5:**
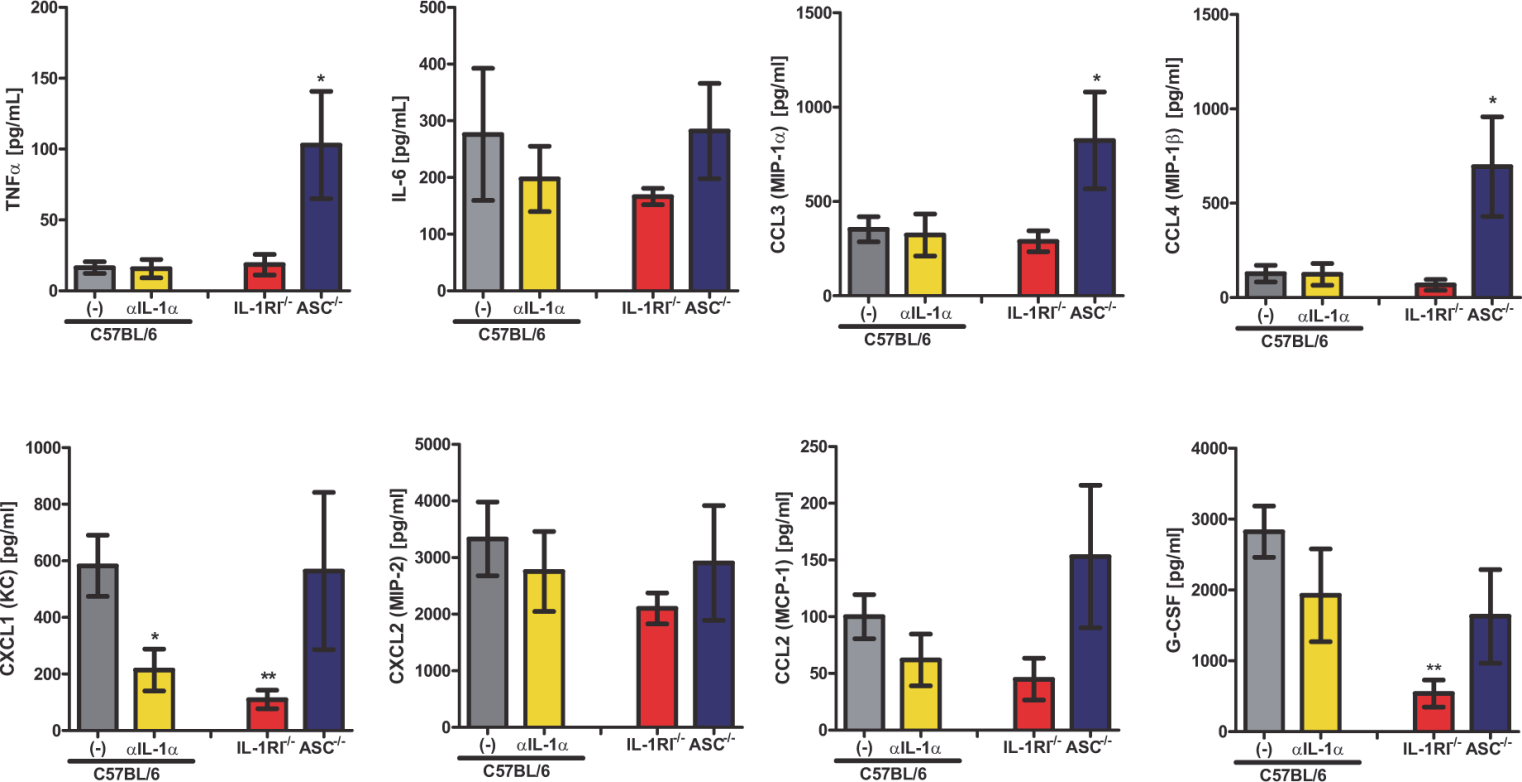
IL-1α signaling enhances expression of leukocyte recruiting chemokines. C57BL/6 mice treated with either isotype control antibody or IL-1α neutralizing antibody, *Il1r1*-deficient and *Pycard*-deficient mice were infected with 5×10^7^ CEA10 conidia and at 24 hours post-infection, mice were euthanized, BALF collected, and lung tissue homogenized. Cytokine and chemokine levels in the lung homogenates were measured using 12-plex multiplex Luminex assay, similar trends were observed in BALF. Data are representative of two independent experiments consisting of 4–5 mice per group. Bar graphs show the group mean ± one SEM. Statistically significant differences were determined using a Kruskal-Wallis one-way ANOVA with Dunn’s post-test (*p < 0.05, **p < 0.01).

As both *Il1r1*-deficient mice and anti-IL1α treated C57BL/6 mice displayed significantly decreased cellular infiltration into the BALF (Fig. 2D and 4C), we next sought to understand the roles that IL-1α, IL-1RI, and the inflammasome play in setting up the inflammatory milieu within the lungs. Thus, we challenged four cohorts of mice, C57BL/6 treated with 40 μg of goat IgG, C57BL/6 treated with 40 μg of anti-IL1α, *Il1r1*-deficient, and *Pycard*-deficient, with ∼5×10^7^ conidia of *A. fumigatus*. Twenty-four hours after challenge, the inflammatory milieu in the lung parenchyma was assessed by a 12-plex multiplex cytokine assay. Anti-IL1α treatment, rather than *Pycard*-deficiency, largely mirrored the inflammatory cytokine defects found in the *Il1r1*-deficient mice (Fig. 5) fitting with the biological outcomes of *A. fumigatus* challenge in those mice. Specifically, TNFα, CCL3, and CCL4 expression was not diminished in the absence of IL-1α, IL-1RI, or ASC (Fig. 5). Interestingly, CXCL1 and G-CSF expression were significantly reduced in *Il1r1*-deficient mice. CXCL1 expression was almost entirely dependent on IL-1α signaling (Fig. 5), while G-CSF expression trend to being dependent on both IL-1α and ASC in an additive manner (Fig. 5). A similar trend, as observed with CXCL1, was seen with IL-6 and CCL2, but it did not reach significance (Fig. 5). Thus, blocking IL-1α in mice significantly decreased the abundance of CXCL1 in the lungs, which correlates with the decreased neutrophil recruitment in *Il1r1*-deficient mice.

### Absence of CCR2^+^ monocytes results in decreased IL-1α, CXCL1, and neutrophil recruitment

As IL1α was necessary for optimal CXCL1 expression and neutrophil infiltration into the BALF (Fig. 4C & 5), we next sought to identify potential cellular sources of IL-1α in response to pulmonary challenge with *A. fumigatus*. Within the lung of a naïve mouse several potential sources of IL-1α exist including: non-hematopoietic cells (epithelial and endothelial cells), alveolar macrophages in the airway spaces, and CCR2^+^ monocytes within the lung parenchyma. During pulmonary *Mycobacterium tuberculosis* infection two distinct populations of myeloid cells co-express IL-1α and IL-1β: inflammatory monocytes which are CD11b^+^ CD11c^−^ Ly6c^+^ and monocytic dendritic cells which are CD11b^+^ CD11c^+^ [49]. In response to pulmonary *A. fumigatus* challenge, CCR2^+^ inflammatory monocytes are rapidly recruited to the lung and give rise to monocyte-derived dendritic cells that play essential roles in innate defense against invasive aspergillosis [12]. Interestingly, both CCR2^+^ inflammatory monocytes and monocyte-derived dendritic cells show increased transcription of the *Il1a* gene at 48 h post-*A.fumigatus* challenge [12], but whether lung-resident CCR2^+^ monocytes could contribute to IL-1α production at early times after infection was not explored.

**Figure 6:**
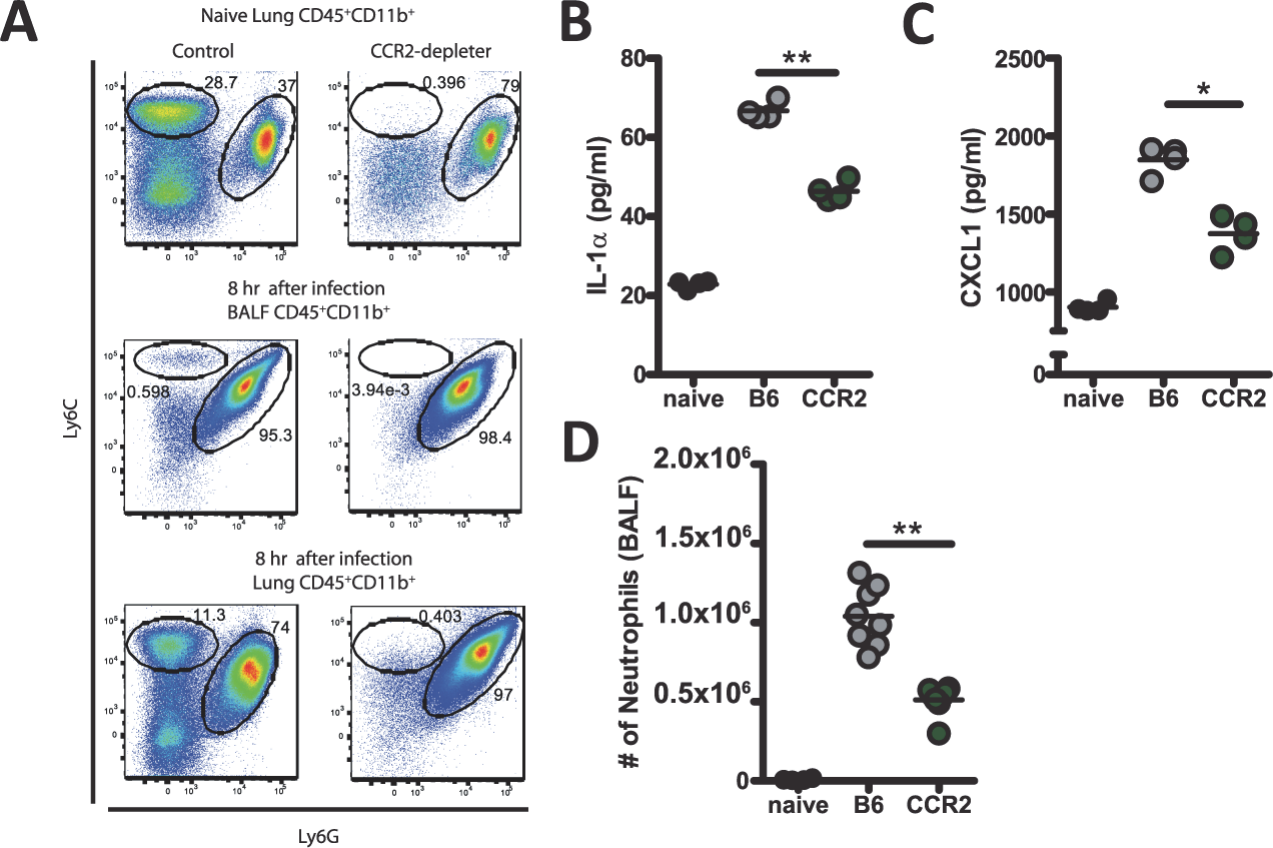
CCR2^+^ monocyte regulate early IL-1α and CXCL1 expression. C57BL/6 or CCR2-depleter mice were treated i.p. with 250 ng of DT 24 h prior to challenge with 5×10^7^ Af293 conidia. **(A)** Naïve C57BL/6 or CCR2-depleter mice or C57BL/6 or CCR2-depleter mice challenged eight hours prior were euthanized and the BALF and lung tissue collected for flow cytometric analysis to assess depletion of target cells by DT. Plots are gated on CD45^+^ CD11b^+^ cells and show Ly6c and Ly6g staining, which identify the CCR2^+^ monocytes and neutrophils, respectively. **(B)** IL-1α and **(C)** CXCL1 protein levels in the lung parenchyma at 8 h post-challenge with 5×10^7^ conidia of *A. fumigatus* strain Af293 were measured using ELISA assays. Bar graphs show the group means ± one SEM. **(D)** Eight hours post-challenge with 5×10^7^ conidia of *A. fumigatus* strain Af293, neutrophils in the BALF were enumerated. Data are representative **(B-C)** or pooled **(D)** from two independent experiments consisting of 4 mice per group. Each symbol represents an individual mouse and the line represents the group mean. Statistically significant differences were determined using a one-way ANOVA with Bonferroni’s post-test compared C57BL/6 mice (*p < 0.05, **p < 0.01).

Thus, we challenged either C57BL/6 or CCR2-depleter mice [10,50], which had been treated one day prior with 250 ng of diphtheria toxin, with ∼5×10^7^ conidia of *A. fumigatus*. To confirm depletion of the CCR2^+^ monocytes we quantified CCR2^+^ inflammatory monocytes (identified as CD45^+^CD11b^+^Ly6C^+^Ly6G^−^) in the BALF and lung parenchyma 8 h after *A. fumigatus* challenge by flow cytometry. We found that diphtheria toxin had no effect on CCR2^+^ monocytes in control animals, while CCR2-depleter mice treated with DT had no detectable Ly6C^+^ inflammatory monocytes, in the BALF or lung parenchyma as expected (Fig. 6A) [12]. We found that IL-1α protein levels were significantly decreased when CCR2^+^ monocytes were absent (Fig. 6B) consistent with the idea that lung-resident CCR2^+^ inflammatory monocytes are important for producing and/or inducing expression of this cytokine in the lung at early times after infection. Since blocking IL-1α in mice significantly decreased the expression of CXCL1 in the lungs (Fig. 5), we next asked whether CXCL1 protein levels were diminished in the lung parenchyma of the CCR2-depleter mice. We found that CXCL1 protein levels were also significantly decreased in the absence of CCR2^+^ monocytes (Fig. 6C). Thus, CCR2^+^ monocytes are important regulators of the early expression of IL-1α and CXCL1. Consistent with the importance of these factors in promoting early neutrophil recruitment (Fig. 2 and 4), diminished IL-1α and CXCL1 levels in CCR2-depleter mice correlated with diminished recruitment of neutrophils to the airways 8 h after *A. fumigatus* challenge (Fig. 6D). Thus, CCR2^+^ monocytes are important regulators of the early expression of IL-1α and CXCL1, which are required for optimal recruitment of neutrophils at early times after *A. fumigatus* challenge.

### CXCL1 supplementation of *Il1r1*-deficient mice enhances neutrophil recruitment and resistance to pulmonary *Aspergillus fumigatus* challenge

**Figure 7:**
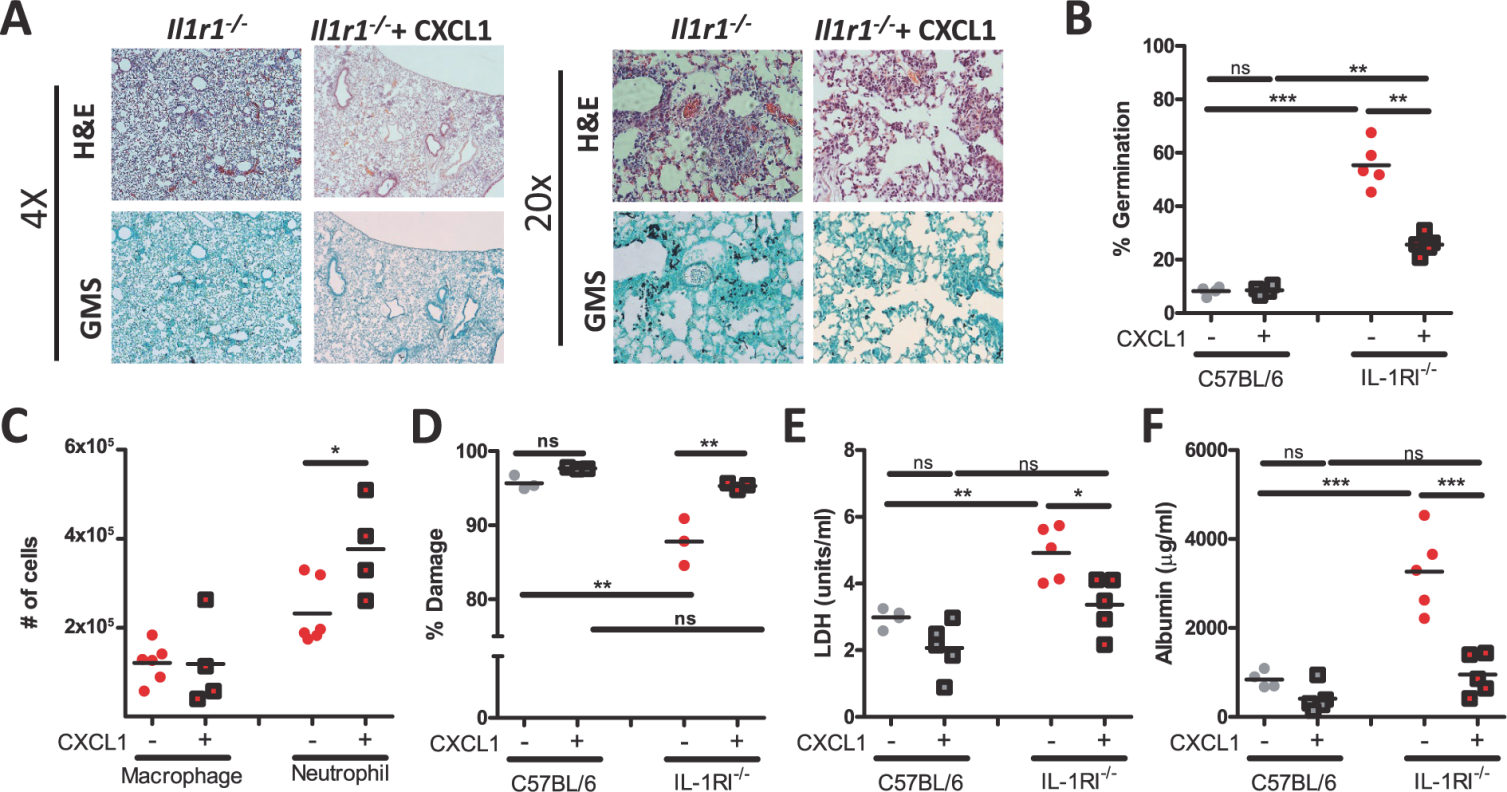
Treatment of *Il1r1*-deficient mice with CXCL1 partially increases resistance to *Aspergillus fumigatus* infection. C57BL/6 mice and *Il1r1*-deficient mice were challenged i.t. with 5×10^7^ CEA10 conidia. Three hours post-challenge mice were given 0.5 μg CXCL1 i.t. or PBS alone. Twenty-four hours post-infection, mice were euthanized, BALF collected, and lungs saved for histological analysis. **(A)** Formalin-fixed lungs were paraffin embedded, sectioned and stained with H&E (top) or GMS (bottom) for analysis by microscopy. Representative lung sections from *Il1r1*-deficient mice challenged with CEA10 for 48 h and treated with either PBS or CXCL1 are shown using either the 4× (left) or 20× (right) objectives. **(B)** *A. fumigatus* germination rates were assessed at 48 h of infection by microscopically counting both the number of conidia and number of germlings in GMS-stained section. Number of conidia and number of germlings were counted for each GMS-stained section to quantify the percent germination. **(C)** Macrophage and neutrophil recruitment in *Il1r1*-deficient mice 24 h post-challenge infected with *A. fumigatus* treated with PBS or CXCL1 given i.t. was determined via cytospins.**(D)** Bone marrow neutrophils from C57BL/6 and *Il1r1*-deficient mice were incubated with CEA10 germlings *in vitro* at a 10:1 ratio in normoxia for 2 h. The XTT assay was used to determine percent fungal damage. **(E)** Lung damage and **(F)** leakage were assessed by measuring LDH and albumin, respectively. Data is representative of at least two independent experiments consisting of three to five mice per group, except for the bone marrow neutrophil anti-hyphal XTT assay which is a single experiment which consisted of pooled bone marrow neutrophils from three mice done in triplicate. Each symbol represents an individual mouse or replicate and the line represents the group mean. Statistically significant differences were determined using a one-way ANOVA with Bonferroni’s post-test (*p < 0.05, **p < 0.01, ***p < 0.001, ns = not significant).

As both *Il1r1*-deficient mice and anti-IL1α treated mice displayed significantly decreased cellular infiltration into the BALF (Fig. 2D and 4C) that correlated with decreased abundance of CXCL1 (Fig. 5), we next sought to test whether immunotherapy which enhances neutrophil accumulation in the lungs, such as CXCL1 supplementation, could enhance control of *A. fumigatus* growth in the *Il1r1*-deficient mice. We challenged either C57BL/6 or *Il1r1*-deficient mice with ∼5×10^7^ conidia of *A. fumigatus*. Three hours after challenge mice were treated i.t. with either PBS or 0.5 μg CXCL1. As expected, *Il1r1*-deficient mice displayed a significant impairment in controlling *A. fumigatus* germination at 48 h when compared with C57BL/6 mice (Fig. 7A-B). Provision of CXCL1 to *Il1r1*-deficient mice could partially rescue control of *A. fumigatus* germination in the lungs, while no enhancement in control of fungal growth was observed in the CXCL1 treated C57BL/6 mice (Fig. 7A-B). Furthermore, provision of CXCL1 i.t. rescued the impairment of anti-IL1α treated C57BL/6 mice in controlling *A. fumigatus* infection (S5A Fig.). As expected, twenty-four hours after challenge the recruitment of neutrophils, but not macrophages, to the BALF in *Il1r1*-deficient mice was enhanced by the provision of CXCL1 (Fig. 7C). Additionally, neutrophil recruitment to the BALF in anti-IL1α treated C57BL/6 mice was significantly enhanced (S5B Fig.). While CXCL1 provision enhanced neutrophil accumulation in the airways of *Il1r1*-deficient mice, we also sought to test whether the anti-hyphal activity of neutrophils was altered in the absence of IL-1RI signaling but exogenous addition of CXCL1. Hyphal damage induced by neutrophils isolated from the bone marrow of respective mouse genotypes was assessed using the XTT hyphal damage assay [46]. C57BL/6 bone marrow neutrophils induced robust hyphal damage when co-cultured with *A. fumigatus*, which was not further enhanced by treated with 50 nM of CXCL1 (Fig. 7D). Interestingly, *Il1r1*-deficient bone marrow neutrophils induced significantly less damage to *A. fumigatus* hyphae than was observed with C57BL/6 bone marrow neutrophils (Fig. 7D). In contrast to the treatment of C57BL/6 bone marrow neutrophils, treatment of *Il1r1*-deficient bone marrow neutrophils with 50 nM of CXCL1 significantly enhanced the anti-hyphal activity of those cells (Fig. 7D). When cell death and endothelial/epithelial cell leakage were assessed *in vivo* by lactate dehydrogenase (LDH) and albumin measurement, respectively, in the BALF both markers were increased in the absence of IL-1RI (Fig. 7E-F). CXCL1 supplementation reduced both markers, but albumin levels were more dramatically reduced than LDH levels (71% versus 32%, respectively) (Fig. 7E-F). Thus, provision of CXCL1 could significantly enhance neutrophil recruitment to the lungs in the absence of IL-1α signaling and enhanced the *in vitro* anti-hyphal activity of *Il1r1*-deficient bone marrow neutrophils, which together ultimately resulted in a partial repair of the *A. fumigatus* control mechanisms in the *Il1r1*-deficent lungs.

## Discussion

In this study, we uncover an essential function for IL-1RI in preventing fungal proliferation and host damage in murine lungs. We have demonstrated a novel dichotomy for the IL-1 cytokines in regulating the innate immune response induced by *A. fumigatus*. Specifically, IL-1α is required for initiating the correct inflammatory signals necessary for optimal leukocyte recruitment, while the inflammasome and IL-1β was necessary for optimal anti-fungal activity against fungal hyphae. We have elucidated that IL-1α plays the dominant role in activating IL-1RI signaling which results in amplified CXCL1 expression, which correlated with optimal leukocyte recruitment to the respiratory tract. CCR2^+^ monocytes were important cells in regulating the early production of IL-1α, CXCL1, and neutrophil recruitment. Taken together, our data demonstrate that signaling through IL-1RI by both IL-1α and IL-1β was necessary for optimal control of *A. fumigatus* pulmonary challenge to prevent IPA development.

IL-1RI signaling was essential in resisting pulmonary *A. fumigatus* challenge in our studies, as demonstrated by *Il1r1*-deficient mice being unable to resist fungal growth resulting in significant mortality in those animals. This finding is consistent with results reported by Pearlman and colleagues who also found that *Il1r1* was needed to prevent the development of *A. fumigatus* induced keratitis [51]. Moreover, van de Veerdonk and colleagues have recently shown that a polysaccharide fungal virulence factor, galactosaminogalactan (GAG), from *A. fumigatus* induces the expression of IL-1Ra, which antagonizes IL-1 signaling resulting in enhanced susceptibility to IPA [52]. Gresnigt *et al* demonstrated that GAG pretreatment of BALB/c mice resulted in more fungal growth associated with impaired neutrophil recruitment, which was completely dependent on IL-1Ra expression [52]. However, the importance of GAG induction of IL-1Ra during a live pulmonary *A. fumigatus* infection remains unknown. GAG expression might actually be reduced at specific infection sites during *in vivo A. fumigatus* challenge because hypoxia, which occurs after *A. fumigatus* challenge [47] has been observed to reduce GAG production [46]. In general, the temporal and spatial dynamics of fungal cell wall PAMPs *in vivo* during an active infection is not fully understood and likely complicated by the heterogeneous nature of the lung and infection site microenvironments.

Downstream of IL-1RI the proximal signaling adapter to propagate IL-1 signaling is MyD88. Similar to our observation with *Il1r1*-deficient mice, Marr and colleagues [44] and Hohl and colleagues (Jhingran A. *et al*, in press) have found the *Myd88*-deficient mice are more susceptible to *A. fumigatus* challenge. Additionally, impaired control of pulmonary histoplasmosis and disseminated candidiasis was observed in *Il1r1*-deficient and *Il1a/Il1b*-doubly deficient mice, respectively [41,45]. While our studies and the studies just discussed strongly support a role for IL-1RI signaling in limiting IPA, and other invasive fungal infections, Romani and colleagues have observed that *Il1r1*-deficient mice were more resistant to pulmonary *A. fumigatus* challenge [53]. The difference with our study is potentially due to the *A. fumigatus* strain studied, as Romani and colleagues have shown that different *A. fumigatus* strains have diverse abilities to induce pathology and immune responses [54]. Importantly, the infection models studied are significantly different, with Romani and colleagues utilizing a cyclophosphamide-induced immunosuppression model with *Aspergillus* conidia delivered on 3 consecutive days intranasally [53], while our studies used immunocompetent mice and a single dose of *Aspergillus* conidia given intratracheally. Additionally Bellocchio et al. [53], reported that histological analyses in the *Il1r1*-deficient mice revealed “numerous fungal elements in the relative absence of signs of inflammatory pathology” which is consistent with the results we report here in our experimental model. Murine mortality in infectious disease models can result from direct pathogen mediated damage or immunopathogenesis, and it is unclear, in this regard, how our models differ. What appears to be clear, however, is that in the absence of IL-1RI signaling, *Aspergillus* proliferation increases *in vivo*. Taken together, all these findings demonstrate that the IL-1 signaling pathway is likely central for resistance to fungal diseases, but their role during immunosuppression and frequency of fungal exposure/quantity may differ, warranting further exploration of the IL-1 cytokine family in each clinically relevant model of IPA.

In further support of our observation, the protective role of IL-1 cytokines in anti-fungal immunity uncovered in our study using the murine model of *A. fumigatus* infection is likely to be operational in humans as indicated by genetic linkage studies. First, individuals with SNPs in the IL-1 gene cluster, which are associated with decreased IL-1 dependent inflammatory events, were at increased risk for the development of IPA [25,26]. Second, polymorphisms in the *CIAS1* gene play a central role regulating inflammasome activity and IL-1β production, which can alter the risk of a subset of patients to developing recurrent vulvovaginal candidiasis [55]. Third, macrophages from patients with chronic cavitary pulmonary aspergillosis (CCPA) had prolonged expression of *Il1a* and *Il1b* after *A. fumigatus* treatment when compared to healthy controls and SNPs in the *Il1b* and *Il1rn* loci are associated with susceptibility to developing CCPA [56]. Thus, targeting the IL-1 cytokine pathways in humans could be important in managing fungal infections.

In previous papers exploring the role of MyD88 during *A. fumigatus* [44,51] it was shown that fungal growth was not controlled, but the mechanism impaired in the absence of IL-1RI and MyD88 signaling remains an open question. In this study and a parallel study by Jhingran *et al* (in press) it was demonstrated that MyD88 and IL1RI mediated signals are necessary for optimal leukocyte recruitment after pulmonary *A. fumigatus* challenge, which is needed for preventing the development of IPA. Analogously, in the *A. fumigatus* keratitis model both *Myd88*- and *Il1r1*-deficient mice demonstrated reduced cellular infiltrate early after inoculation [51]. However, why the lack of IL-1RI or MyD88 signaling results in decreased cellular infiltrates was an open question. Interestingly, we found a decrease in the expression of the chemokine CXCL1 in *Il1r1*-deficient mice, which others have also observed in challenged *Myd88*-deficient mice [44] (Jhingran A. *et al*, in press). CXCL1, together with CXCL2 and CXCL5, are ligands for CXCR2 and are key chemoattractants for recruitment of neutrophils. Administration of a blocking anti-CXCR2 antibody or genetic ablation of *Cxcr2* has been shown to exacerbate mortality and delay neutrophil recruitment following pulmonary *A. fumigatus* challenge [23,24]. Moreover, transient over-expression of CXCL1 in CC10-expressing lung epithelial cells resulted in significantly enhanced leukocyte accumulation and reduced fungal burden [22]. Correspondingly, when we treated *Il1r1*-deficient mice with recombinant murine CXCL1 we observed significantly enhanced neutrophil accumulation. In addition, *Il1r1*-deficient bone marrow neutrophils displayed decreased anti-hyphal activity *in vitro*, which was restored by treatment with CXCL1. These data demonstrate that both IL-1RI and CXCL1 signaling is critical in not only enhancing neutrophil recruitment to the airways in the *Il1r1*-deficient mice, but also in inducing the optimal anti-hyphal state of the recruited neutrophils. While the mechanism behind CXCL1 mediated anti-hyphal activity in our model is unknown, neutrophils from *Cxcl1*-deficient mice have an impaired reactive oxygen response in a polymicrobial sepsis model [57]. Moreover, *Cxcl1*-deficient neutrophils stimulated with *Klebsiella pneumoniae* had reduced expression of p67^*phox*^ and p47^*phox*^ and reduced production of myeloperoxidase, nitric oxide, and hydrogen peroxide, which results in decreased killing of *Klebsiella pneumoniae* by the neutrophils [58]. Finally, IL-8 has been shown to be important for priming the human neutrophils reactive oxygen burst [59]. Thus, our data supports a model where IL-1RI signaling is critical for optimal neutrophil recruitment and activation of their anti-hyphal activity in part through the regulation of CXCL1 abundance. Further support for this conclusion comes from a recent analysis of mice with a myeloid deficiency of the transcriptional regulator HIF1α. Loss of myeloid HIF1α results in severe susceptibility to the same strain of *A. fumigatus* utilized here in part through reduction in neutrophil recruitment. Importantly, loss of HIF1α resulted in decreased IL1-α and CXCL1 levels after *A. fumigatus* challenge similar to what we observed in our studies [60]. Interestingly, other inflammatory pathways are also temporally regulating neutrophil recruitment after *A. fumigatus* challenge, because *Card9*-deficient mice had a late defect in neutrophil recruitment that was associated with a more global diminution of the inflammatory milieu [61]. In addition to the early defect in neutrophil recruitment *Il1r1*-deficient mice also had decreased macrophage recruitment to the airways by 48 h post-inoculation. The reason for this is unknown at this time, but it is known that G-CSF deficient mice have monocyte defects and our cytokine analysis demonstrated that *Il1r1*-deficient mice had significantly lower level of G-CSF in the airways [62]. Thus, further studies exploring the regulation of multiple neutrophil chemotactic pathways, such as CXCR2-, CCR1-, IL17-, leukotriene-, and complement-dependent pathways, and monocyte chemotactic pathways, such as G-CSFR—and CCR2-dependent pathways, are needed after pulmonary fungal challenge.

IL1RI, together with IL1RAcP, is the high-affinity receptor for both IL-1α and IL-1β [27]. The maturation and secretion of IL-1α and IL-1β is known to be regulated by distinct proteolytic pathways dependent on calpain and caspase-1, respectively [27,63]. Numerous fungal pathogens have been shown to activate the inflammasome resulting in the production of IL-1β [30,31,32,33,34,35,36,37]. Importantly for our studies, others have demonstrated that the NLRP3-ASC-Caspase1 inflammasome could be activated by *A. fumigatus* [31], but the *in vivo* relevance of that finding was unknown. Control of *C. albicans* infection, which also activated the NLRP3-ASC inflammasome, was highly dependent on NLRP3 and IL-1β [30,36,37,41]. In sharp contrast, our current results indicate that the inflammasome only plays a modest role in the control of pulmonary *A. fumigatus* growth. In our experiments, neutrophil recruitment in mice lacking the inflammasome was completely normal, which is in contrast to *C. albicans* infection where mice deficient in IL-1β displayed a significant reduction in neutrophil recruitment [41]. Furthermore, antibody blockade of IL-1β during pulmonary *Histoplasma capsulatum* infection resulted in decreased survival associated with decreased recruitment of Gr-1^+^ cells early and CD4^+^ cells late to the lungs of challenged animals [45]. Thus, we were surprised to observe such a dominant role for IL-1α in regulating early leukocyte recruitment following pulmonary *A. fumigatus* challenge, which correlates with its regulation of the chemokine CXCL1. In support of this finding, during sterile inflammation the importance of IL-1α in regulating neutrophil recruitment is unquestionable [38,39]. It has also been demonstrated that IL-1α plays a critical role during murine *L. pneumophila* infection, initiating neutrophil recruitment and the inflammatory response early after infection [40]. Others have previously shown that IL-1RI and MyD88 expression within a radioresistant population of cells was essential for optimal expression of CXCL1 and CXCL2 during *L. pneumophila* infection [64]. Interestingly, in their parallel study Hohl and colleagues found that IL-1RI/MyD88 signaling in a radioresistant cell population was necessary for optimal CXCL1 expression and neutrophil recruitment early after pulmonary *A. fumigatus* challenge (Jhingran A. *et al*, in press).

Because of the early importance of IL-1 cytokines in regulating the pulmonary anti-fungal immune response, non-hematopoietic cells (epithelial or endothelial cells) or lung-resident myeloid cells could represent potential sources of IL-1α and IL-1β after *A. fumigatus* challenge. During pulmonary *Mycobacterium tuberculosis* infection two distinct populations of myeloid cells co-express IL-1α and IL-1β: inflammatory monocytes which are CD11b^+^ CD11c^−^ Ly6c^+^ and monocytic dendritic cells which are CD11b^+^ CD11c^+^ [49]. After pulmonary *A. fumigatus* challenge both inflammatory monocytes and monocytic dendritic cells are found in the lung parenchyma and both show increased transcription of the *Il1a* gene [12]. Here our data demonstrates that CCR2^+^ monocytes are at least one of the important cell types regulating the early expression of IL-1α and CXCL1, as well as neutrophil recruitment at 8 hpi. However, in the absence of CCR2^+^ monocytes there is still a significant amount of IL-1α and CXCL1 produced in the lungs after *A. fumigatus* challenge, thus there are likely multiple sources of IL-1α that can regulate early pulmonary neutrophil accumulation. Moreover, by 48 h after infection CXCL1 levels and neutrophil recruitment to the lung is unaffected in CCR2-depleter mice [12], thus suggesting that distinct mechanisms of neutrophil recruitment are operational at various times after infection. This is supported by observation that *Myd88*-deficient and *Card9*-deficient mice have early or late defects in neutrophil recruitment, respectively (Jhingran A. *et al*, in press and [61]).

In our experiments the inflammasome and IL-1β appear to regulate the anti-fungal activity of macrophages against hyphae, especially under hypoxic conditions. This enhancement of anti-fungal activity in hypoxic microenvironments is physiologically and clinically important because hypoxia can be generated within the lungs of mice with IPA [47], which is coincident with inflammatory monocyte arrival to the lungs [12]. Understanding how hypoxia can enhance the anti-fungal activity of macrophage in an inflammasome and IL-1β dependent manner will be important in understanding how macrophages limit fungal growth. Interestingly, a recent paper from Torres *et al* demonstrated that acidosis, which can be driven by hypoxia, resulted in increased IL-1β production in response to *P. aeruginosa* challenge [65]. Perhaps somewhat surprisingly, in the absence of MyD88 anti-fungal activity against *A. fumigatus* conidia remains intact (Jhingran A. *et al*, in press). It has been shown in other fungal pathogens that IL-1β treatment of human peripheral blood leukocytes enhances their anti-fungal activity against *Paracoccidiodies brasiliensis* [66,67]. Additionally, *Il1r1*- and *Nlrp3*-deficient macrophages have impaired antifungal activity against *P. brasiliensis* [34]. In contrast, during disseminated candidiasis *Il1a*-deficiency was associated with decreased anti-fungal activity of leukocytes [41]. Thus, studies designed to understand the differential dependencies of the IL-1 cytokines in regulating leukocyte recruitment and anti-fungal activity during a range of fungal diseases and morphological forms are needed.

In addition to understanding the cellular source of IL-1α and IL-1β, understanding the inflammatory pathways leading to expression of IL-1α and IL-1β are essential to our understanding of resistance to IPA. In the absence of dectin-1 signaling there is decreased expression of both IL-1α and IL-1β [68,69]. The loss of HIF1α in the LysM-expressing cells also resulted in decreased IL-1α levels after *A. fumigatus* challenge [60], which can be regulated by dectin-1 agonists such as β-glucan [70]. Pulmonary *A. fumigatus* infection results in significant tissue damage and cell death, but the exact type of cell death is not known. Moreover, the phenotype of cell death will be shaped by the hypoxic microenvironment found during IPA. The type of cellular death occurring *in vivo* during *A. fumigatus* will have important immunological impacts shaping the early IL-1α and IL-1β response because necrotic cell death favors IL-1α release while pyroptosis favors IL-1β release [71]. Interestingly, *C. albicans* mutants with defects in inducing pyroptosis also demonstrated defects in inducing IL-1β secretion, but IL-1α release was not examined [72]. Additionally, understanding how deficiencies in PRR signaling alters the overall inflammatory response will be crucial as patients with SNPs in PRRs are known to have elevated risks for developing IPA [73]. Our data demonstrate that in the *Pycard*-deficient mice there are elevated levels of TNFα, CCL3, and CCL4. One explanation for observing elevated levels of TNFα, CCL3, and CCL4 could be the range or degree that PRRs are being engaged in the *Pycard*-deficient mice and/or temporal and spatial dynamics of fungal cell wall PAMP engagement with PRRs *in vivo* that are not fully understood and further complicated by a PRR known to be engaged during infection now being absent.

It is well defined that prolonged corticosteroid treatment increases susceptibility of hosts to IPA [74]. Interestingly, dexamethasone induces the expression and release of IL-1RII [75]. IL-1RII is known to limit the activity of IL-1 cytokines and/or sequester IL-1α protein in the cytosol, preventing the cleavage of IL-1α by calpain [63]. Dexamethasone has also been shown to impair IL-1α and IL-1β secretion from human mast cells in response to *Pseudomonas aeruginosa* stimulation [76]. Moreover, dexamethasone treatment of bronchoalveolar macrophages prior to treatment with *A. fumigatus* conidia significantly impaired their release of IL-1α [77,78]. Because we have uncovered such a prominent role for IL-1α in controlling pulmonary *A. fumigatus* challenge, future studies exploring the cleavage status of IL-1α and expression of IL-1RII in clinically relevant models are critical.

Finally, the importance of appropriate activation of leukocytes in the control of *A. fumigatus* is highlighted by patients with chronic granulomatous disease being highly susceptible to *A. fumigatus* [15,16,17]. Interestingly, CGD patients or mice are typically in a hyperinflammatory state, which is linked to inflammasome activity and IL-1β expression [79,80]. Further, blockade of IL-1 cytokines in p47^*phox*^-deficient mice through treatment with hIL1ra results in improved control of *A. fumigatus* [79]. Together, these studies demonstrate that further exploration of the positive and negative regulators of IL-1 signaling during invasive fungal infections is needed. Moreover, it is critical that we continue to explore the regulation of the inflammatory response induced in each of the different subpopulations of hosts susceptible to developing invasive fungal infections in order to develop patient specific novel immunotherapeutic approaches that could complement treatment with anti-fungal agents.

## Materials and Methods

### Mice

C57BL/6J mice were bred in-house. *Pycard* (ASC)-deficient and *Myd88*-deficient mice were originally provided by Dr. Vishiva Dixit (Genentech) and Dr. Mark Jutila (Montana State University), respectively. *Il1r1*-deficient (Stock #003245) and C57BL/6 (Stock #000664) mice were originally purchased from Jackson Laboratories. Mouse strains were then bred in-house. The CCR2-depleter (CCR2-DTR) strain was generated on the C57BL/6 background as previously described [10,50]. Control animals for CCR2^+^ monocyte depletion experiments were sex—and age-matched, non-transgenic littermates. All mice were 8–10 weeks of age at the time of infection. All animal experiments were approved by the Montana State University or Rutgers University Institutional Animal Care and Use Committee.

### Preparation of *Aspergillus fumigatus* conidia

*A. fumigatus* strain CEA10 or Af293 was grown on glucose minimal media (GMM) agar plates for 3 days or Sabouraud dextrose agar (SDA) for 7–10 days at 37°C, respectively. Conidia were harvested by adding 0.01% Tween 80 to plates and gently scraping conidia from the plates using a cell scraper. Conidia were then filtered through sterile Miracloth, were washed and resuspended in phosphate buffered saline (PBS), and counted on a hemacytometer.

### *Aspergillus fumigatus* challenge pulmonary model

Mice were challenged with *A. fumigatus* conidia by the i.t. route. Mice were anesthetized with 2.5% 2,2,2-tribromoethanol or a Ketamine/Xylazine solution given i.p.; subsequently, mice were challenged i.t. with ∼5 × 10^7^ *A. fumigatus* conidia in a volume of 100 μl. At the indicated time after *A. fumigatus* challenge, mice were euthanized using a lethal overdose of pentobarbital. Bronchoalveolar lavage fluid (BALF) was collected by washing the lungs with 2 ml of PBS containing 0.05*M* EDTA. BALF was clarified by centrifugation and stored at −20°C until analysis. BAL cells were resuspended in 200 μl of PBS and total BAL cells were determined by hemacytometer count. BAL cells were subsequently spun onto glass slides using a Cytospin4 cytocentrifuge (Thermo Scientific) and stained with Diff-Quik stain set (Siemens) for differential counting. For histological analysis lungs were filled with and stored in 10% buffered formalin phosphate for at least 24 hours. Lungs were then embedded in paraffin and sectioned into 5-micron sections. Sections were stained with H&E and GMS using standard histological techniques to assess lung inflammatory infiltrates and fungal germination, respectively. For cytokine analysis lungs were homogenized in 2 ml of PBS. After clarification, lung homogenates were stored at −20°C until analysis.

### Neutralizing antibodies and chemokine reconstitution

For IL-1α neutralization studies, normal goat IgG control and anti-mIL-1α neutralizing antibody were purchased from R&D systems. IgG control or anti-mIL-1α neutralizing antibody were administered i.p. at 40 μg per mouse. Administration of neutralizing antibody was given every other day, beginning the day prior to *A. fumigatus* challenge. For CXCL1 reconstitution studies, recombinant murine CXCL1 was purchased from PeproTech. CXCL1 was administered i.t. at 0.1–0.5 μg per mouse and was given 3 hours after *A. fumigatus* challenge. For the hIL1ra studies, recombinant hIL1ra and the appropriate placebo (Amgen) were kindly provided by Dr. Charles A. Dinarello. The hIL1ra and placebo were administered i.p. at 200 mg per mouse given at −24, 0, and +24 h relative to *A. fumigatus* challenge.

### CCR2^+^ cell depletion strategy

For depletions of CCR2^+^ cells, CCR2-DTR mice and control littermates received 250 ng of diphtheria toxin i.p. one day prior to infection. Diphtheria toxin was purchased from List Biological Laboratories (Campbell, CA) and reconstituted at 1 mg/ml in PBS. Aliquots were stored at −80°C. The specificity and efficiency of depletion in the lung was confirmed by flow cytometry.

### Quantification of lung damage and leakage

To assess lung damage, bronchoalveolar lavage fluid was analyzed by measuring lactate dehydrogenase levels using a CytoTox 96 Cytotoxicity Assay (Promega) following the manufacturer’s instructions. To assess vascular/pulmonary leakage, bronchoalveolar lavage fluid was analyzed using an Albumin BCG Reagent Set (Eagle Diagnostics). A standard curve was made by diluting the calibrator in PBS. Then 100 μl of sample or standard was transferred to a 96 well flat-bottomed plate, mixed with 100 μl of BCG reagent, let sit at RT for 5 min and then read on a plate reader at 630 nm.

### Lung cell isolation and flow cytometric analysis

After collection of the BAL fluid, lung samples were minced in RPMI containing 100 units/ml of collagenase (Gibco) at 37°C for 60 minutes, followed by disruption through a 40-μm filter. After which, red blood cells were lysed using a Tris ammonium chloride solution. Staining of ∼10^7^ cells was performed in 200 μl of PBS containing 2% bovine serum and 2 mM EDTA. For analysis of leukocytes, antibody staining was both conducted at 4°C for 30 minutes. Phenotypic analysis of leukocytes was conducted using a panel of cell surface markers: CD11b, CD11c, Ly6g, Ly6c, 7/4, CD19, and I-A/I-E, as previously described [10]. All antibodies used for analysis were purchased from Biolegend, BD Biosciences, eBioscience or Novus Biologicals. After staining, cells were washed and fixed with 1% paraformaldehyde in PBS. Fluorescent intensities were measured using an LSR (BD Biosciences) and data were analyzed using FlowJo software (Tree Star).

### Assay for cytokine, chemokine, and soluble receptor secretion

Bronchoalveolar lavage fluid and lung homogenates from C57BL/6 mice challenged with *A. fumigatus* for 6, 12, 24, and 48 h were initially analyzed for cytokines and chemokines using ProcartaPlex Mouse Cytokine & Chemokine 36-plex (Affymetrix-eBioscience). IL-1Ra levels were determined by ELISA (R&D Systems). Plates were read using a BioPlex 200 (Bio-Rad) or a SpectraMax Paradigm plate reader (Molecular Devices).

### Growth of bone marrow-derived macrophages (BMDM)

Femurs and tibias from 8–10 week old mice were obtained and centrifuged to collect bone marrow. Cells were resuspended in media containing RPMI 1640, 2 mM L-glutamic acid, 50 mg/l gentamycin, 100 U/ml penicillin/streptomycin, 30% L929 cell supernatant, 20% FBS and 0.0004% 2-ME. On day 3 fresh medium was added to the cultures. Cells were incubated for a total of 6 days at 37°C and 5% CO_2_.

### Isolation of bone marrow neutrophils

Bone marrow neutrophils were isolated from femurs and tibias from 8–12 week old C57BL/6 and *Il1r1*-deficient mice as previously described [60]. Briefly, single cell suspensions of bone marrow in HBSS containing 0.1% FBS and 1% glucose were resuspended in 3 ml of 45% Percoll (GE Healthcare). A discontinuous Percoll gradient was set-up consisting of (top to bottom) 3 ml 45%, 2 ml 50%, 2 ml 55%, 2 ml 62%, and 3 ml 81%. Gradients were then centrifuged for 30 min at 1600 × *g* in a Sorvall Legend Mach 1.6R benchtop centrifuge. Bone marrow neutrophils were collected from the 62%/81% border and washed with HBSS before counting and viability assessment.

### In vitro fungal damage assay

An XTT assay was used to measure fungal metabolic activity as previously described [46]. Bone marrow derived macrophages and CEA10 germlings were incubated together in normoxic or hypoxic conditions at a 10:1 (effector:target) ratio for 5 hours. Bone marrow neutrophils and CEA10 germlings were incubated together in normoxic conditions at a 10:1 (effector:target) ratio for 2 hours with or without 50 nM CXCL1 [81]. Following incubation, macrophages or neutrophils were lysed and the remaining fungi were incubated with 0.4 mg/ml XTT and 0.05 mg/ml coenzyme Q for 1 h and the optical density (OD) subsequently measured on a spectrophotometer at a wavelength of 450 nm. The percent fungal damage was defined by the equation: (1-[A_450_ of fungi with cells—A_450_ of cells alone] / [A_450_ of fungi alone]) * 100.

### Statistical analysis

Statistical significance was determined by a Student’s t-test, one-way ANOVA using a Bonferroni post-test, or Kruskal-Wallis one-way ANOVA with Dunn’s post-test through the GraphPad Prism 5 software as outlines in the figure legends.

## Supporting Information

S1 Fig hIL-1ra treatment results in impaired control of *Aspergillus fumigatus*. C57BL/6 mice were treated with recombinant hIL-1ra or placebo and infected i.t. with 5×10^7^ CEA10 conidia. Forty-eight hours post-infection, mice were euthanized, BALF collected, and lungs saved for histological analysis. Formalin-fixed lungs were paraffin embedded, sectioned, and stained with GMS for analysis by microscopy. *A. fumigatus* germination rates were assessed at 48 h of infection by microscopically counting both the number of conidia and number of germlings in GMS-stained section. Number of conidia and number of germlings were counted for each GMS-stained section to quantify the percent germination. Data are representative of one experiment consisting of 5 mice per group. The bar graph show the group means ± one SEM. Statistically significant differences were determined using a Student’s t-test (**p < 0.01).
(EPS)

S2 Fig Impaired neutrophil recruitment in the lung parenchyma of *Il1r1*-deficient mice. C57BL/6, *Il1r1*-deficient, and *Pycard*-deficient mice were challenged i.t. with 5×10^7^ CEA10 conidia. At 12, 24, and 36 h post-challenge, mice were euthanized, BALF and lungs collected for flow cytometric analysis of neutrophil, monocyte, and dendritic cell populations with the lungs, like previously done [10]. **(A)** Neutrophils in the lungs were identified as being CD45^+^ Ly6g^+^ 7/4^+^. **(B-E)** Lungs were digested in collagenase to generate single cell suspensions, after which cells were stained for flow cytometric analysis. Lung neutrophils were identified as CD45^+^ CD11b^+^ Ly6g^+^ 7/4^+^ **(B)**, CD11b^+^ macrophages were identified as CD45^+^ Ly6g^−^ CD11b^+^ CD11c^−^ CD103^−^ **(C)**, CD11c^+^ macrophages were identified as CD45^+^ Ly6g^−^ CD11b^+^ CD11c^+^ CD103^−^ **(D)**, CD103^+^ dendritic cells were identified as CD45^+^ Ly6g^−^ CD11b^−^ CD11c^+^ CD103^+^ **(E)**. Each time-point represents 8–10 mice pooled from two independent experiments. Data are presented as box and whisker plots with Tukey whisker and outliers displayed as dots. Statistically significant differences were determined using a one-way ANOVA with Bonferroni’s post-test (*p < 0.05; **p < 0.01).
(EPS)

S3 Fig *Myd88*-deficient mice are impaired in their ability to recruit neutrophils after *Aspergillus fumigatus* challenge. Age-matched C57BL/6 or *Myd88*-deficient mice were infected i.t. with 5×10^7^ CEA10 conidia and at indicated time-points, mice were euthanized, and BALF collected. Total macrophage (left panel) and neutrophil (right panel) recruitment in the BALF was measured at 12 and 24 h post-challenge. Data are representative of at least 2 independent experiments at each time point consisting of 3–5 mice per group. Bar graphs show the group means ± one SEM. Statistically significant differences were determined using Student’s t-test (*p < 0.05; **p < 0.01).
(TIF)

S4 Fig *Pycard*-deficient mice treated with IL-1α neutralizing antibody are highly susceptible to *Aspergillus fumigatus* infection. C57BL/6 or *Pycard*-deficient mice treated with isotype control antibody or IL-1α neutralizing antibody were infected i.t. with 5×10^7^ CEA10 conidia. Twenty-four hours post-infection mice were euthanized, BALF collected and lungs saved for histological analysis. Formalin-fixed lungs were paraffin embedded, sectioned, and stained GMS for analysis by microscopy. *A. fumigatus* germination rates were assessed 48 h after challenge by microscopically counting both the number of conidia and number of germlings in GMS-stained section. Data are representative of two independent experiments consisting of 4–5 mice per group. The bar graph show the group means ± one SEM. Statistically significant differences were determined using a one-way ANOVA with Bonferroni’s post-test (**p < 0.01, ***p < 0.001).
(TIF)

S5 Fig Intratracheally provision of CXCL1 to anti-IL1α treated C57BL/6 mice increases resistance to *Aspergillus fumigatus* infection. C57BL/6 mice were treated with goat IgG or anti-IL1α 24 h prior to and 24 h after i.t. challenge with 5×10^7^ CEA10 conidia. Three hours post-challenge half the anti-IL1α treated mice were given 0.5 μg CXCL1 in PBS or PBS alone given i.t. At 48 h post-infection mice were euthanized, BALF collected, and lungs saved for histological analysis. Formalin-fixed lungs were paraffin embedded, sectioned and stained with GMS for analysis by microscopy. **(A)** *A. fumigatus* germination rates were assessed 48 h after challenge by microscopically counting both the number of conidia and number of germlings in GMS-stained section. **(B)** Total macrophage (left panel) and neutrophil (right panel) recruitment in the BALF was measured at 24 h post-challenge. Data are representative of two independent experiments consisting of 3–5 mice per group. Bar graphs show the group means ± one SEM. Statistically significant differences were determined using a one-way ANOVA with Bonferroni’s post-test (*p < 0.05).
(TIF)
