# Supplementary figures and images for: IL-1α Signaling Is Critical for Leukocyte Recruitment after Pulmonary Aspergillus fumigatus Challenge
Source: PLoS Pathog. 2015 Jan 28;11(1):e1004625. doi: 10.1371/journal.ppat.1004625 (PMC4309569; doi:10.1371/journal.ppat.1004625)

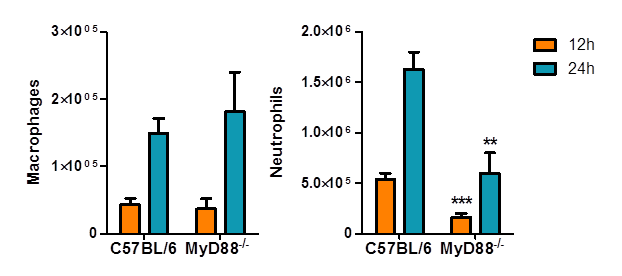

Supplement: S3 Fig — Age-matched C57BL/6 or Myd88-deficient mice were infected i.t. with 5×10^7^ CEA10 conidia and at indicated time-points, mice were euthanized, and BALF collected. Total macrophage (left panel) and neutrophil (right panel) recruitment in the BALF was measured at 12 and 24 h post-challenge. Data are representative of at least 2 independent experiments at each time point consisting of 3–5 mice per group. Bar graphs show the group means ± one SEM. Statistically significant differences were determined using Student’s t-test (*p < 0.05; **p < 0.01). (TIF) [file ppat.1004625.s003.tif]

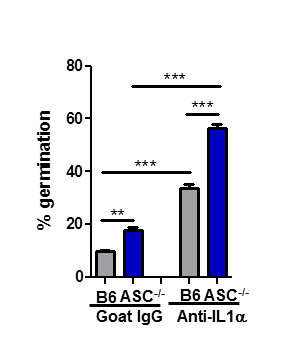

Supplement: S4 Fig — C57BL/6 or Pycard-deficient mice treated with isotype control antibody or IL-1α neutralizing antibody were infected i.t. with 5×10^7^ CEA10 conidia. Twenty-four hours post-infection mice were euthanized, BALF collected and lungs saved for histological analysis. Formalin-fixed lungs were paraffin embedded, sectioned, and stained GMS for analysis by microscopy. A. fumigatus germination rates were assessed 48 h after challenge by microscopically counting both the number of conidia and number of germlings in GMS-stained section. Data are representative of two independent experiments consisting of 4–5 mice per group. The bar graph show the group means ± one SEM. Statistically significant differences were determined using a one-way ANOVA with Bonferroni’s post-test (**p < 0.01, ***p < 0.001). (TIF) [file ppat.1004625.s004.tif]

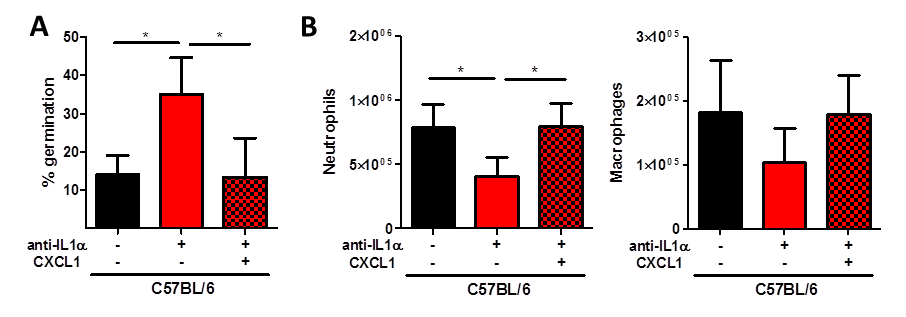

Supplement: S5 Fig — C57BL/6 mice were treated with goat IgG or anti-IL1α 24 h prior to and 24 h after i.t. challenge with 5×10^7^ CEA10 conidia. Three hours post-challenge half the anti-IL1α treated mice were given 0.5 μg CXCL1 in PBS or PBS alone given i.t. At 48 h post-infection mice were euthanized, BALF collected, and lungs saved for histological analysis. Formalin-fixed lungs were paraffin embedded, sectioned and stained with GMS for analysis by microscopy. (A) A. fumigatus germination rates were assessed 48 h after challenge by microscopically counting both the number of conidia and number of germlings in GMS-stained section. (B) Total macrophage (left panel) and neutrophil (right panel) recruitment in the BALF was measured at 24 h post-challenge. Data are representative of two independent experiments consisting of 3–5 mice per group. Bar graphs show the group means ± one SEM. Statistically significant differences were determined using a one-way ANOVA with Bonferroni’s post-test (*p < 0.05). (TIF) [file ppat.1004625.s005.tif]
